# Methylation Profiling Identifies Stability of Isocitrate Dehydrogenase Mutation Over Time

**DOI:** 10.1017/cjn.2023.253

**Published:** 2023-07-12

**Authors:** Mathew R. Voisin, Chloe Gui, Vikas Patil, Andrew F. Gao, Gelareh Zadeh

**Affiliations:** 1 Department of Neurosurgery, Toronto Western Hospital, University of Toronto, Toronto, ON, Canada,; 2 MacFeeters Hamilton Neuro-Oncology Program, Princess Margaret Cancer Centre, University Health Network and University of Toronto, Toronto, ON, Canada; 3 Department of Laboratory Medicine and Pathobiology, Laboratory Medicine Program, University Health Network, University of Toronto, Toronto, ON, Canada

**Keywords:** Gliomas, isocitrate dehydrogenase mutation, glioma evolution, recurrence, methylation profiling

## Abstract

**Objective:**

Isocitrate dehydrogenase (IDH) mutation status is a key diagnostic and prognostic feature of gliomas. It is thought to occur early in glioma tumorigenesis and remain stable over time. However, there are reports documenting a loss of IDH mutation status in a subset of patients with glioma recurrence. Here, we identified patients with a documented loss of IDH mutation status longitudinally and performed multi-platform analysis in order to determine if IDH mutations are stable throughout glioma evolution.

**Methods:**

We retrospectively identified patients from our institution from 2009 to 2018 with immunohistochemistry (IHC)-recorded IDH mutation status changes longitudinally. Archived formalin-fixed paraffin-embedded and frozen tissue samples from these patients were collected from our institution’s tumour bank. Samples were analysed using methylation profiling, copy number variation, Sanger sequencing, droplet digital PCR (ddPCR) and IHC.

**Results:**

We reviewed 1491 archived glioma samples including 78 patients with multiple IDH mutant tumour samples collected longitudinally. In all instances of documented loss of IDH mutation status, multi-platform profiling identified a mixture of low tumour cell content and non-neoplastic tissue including perilesional, reactive or inflammatory cells.

**Conclusions:**

All patients with a documented loss of IDH mutation status longitudinally were resolved through multi-platform analysis. These findings support the hypothesis that IDH mutations occur early in gliomagenesis and in the absence of copy number changes at the IDH loci and are stable throughout tumour treatment and evolution. Our study highlights the importance of accurate surgical sampling and the role of DNA methylome profiling in diagnostically uncertain cases for integrated pathological and molecular diagnosis.

## RÉSUMÉ

**Objectif:**

L’état des mutations de l’isocitrate-déshydrogénase (IDH) est un élément clé du diagnostic et du pronostic des gliomes. On croit que les mutations se produisent au début de la tumorigenèse des gliomes et qu’elles restent stables au fil du temps. Toutefois, la perte de cet état de stabilité des mutations de l’IDH a été documentée dans certains rapports concernant un sous-groupe de patients, au moment de la réapparition des gliomes. Il sera donc question, dans l’article, de patients chez qui la perte de cet état de stabilité des mutations de l’IDH a été documentée longitudinalement, et l’équipe a procédé à une analyse multiplateforme dans le but de déterminer si les mutations de l’IDH étaient stables tout le long de l’évolution des gliomes.

**Méthode:**

Il s’agit d’une étude rétrospective de dossiers de patients repérés dans la base de données de l’établissement des auteurs, de 2009 à 2018, indiquant des changements de l’état des mutations de l’IDH observés à l’immunohistochimie (IHC) et consignés longitudinalement. Des échantillons de tissu fixés au formaldéhyde et enrobés de paraffine (FFPE) et de tissu congelés provenant de ces patients ont été retirés de la banque de tissus tumoraux de l’établissement. Les échantillons ont été analysés à l’aide de diverses techniques : le profilage par méthylation, les variations du nombre de copies, le séquençage selon la méthode de Sanger, l’amplification en chaîne par polymérase numérique en gouttelettes (ddPCR) et l’IHC.

**Résultats:**

Ont été revus 1491 échantillons de gliome conservés, dont ceux de 78 patients présentant des mutations de l’IDH et recueillis longitudinalement. Dans tous les cas documentés de perte de cet état de stabilité des mutations de l’IDH, le profilage multiplateforme a mis en évidence la présence d’un mélange d’un faible contenu en cellules tumorales et de tissu non néoplasique, y compris de cellules périlésionnelles, réactives ou inflammatoires.

**Conclusion:**

L’analyse multiplateforme a permis de résoudre tous les cas de perte de cet état de stabilité des mutations de l’IDH, documentés longitudinalement. Les résultats confirment l’hypothèse selon laquelle les mutations de l’IDH se produisent au début de la gliomagenèse et que, devant l’absence de changement du nombre de copies aux différents locus de l’IDH, celles-ci sont stables tout le long du traitement et de l’évolution des tumeurs. L’étude fait donc ressortir l’importance de la qualité des prélèvements de tissu en cours de chirurgie et le rôle du profilage du méthylome de l’ADN dans les cas d’incertitude diagnostique en vue de l’intégration de résultats histopathologiques et moléculaires en pareille circonstance.

## Introduction

Diffuse gliomas are primary brain tumours that were historically defined by histopathological subtype and graded on a scale of 2–4. With recent molecular advancements, the 2021 World Health Organization Classification of Central Nervous System Tumours describes a classification system that centres on the integration of molecular markers and, in some cases, DNA methylome profiling for the diagnosis and categorization of gliomas.^1^ To date, molecular testing has revealed the clinical and prognostic significance of isocitrate dehydrogenase (IDH) mutation status. IDH mutant gliomas have a distinct biology that is associated with a superior prognosis compared to adult-type gliomas that are IDH wildtype.^2^ Clinically, patients with IDH-mutated tumours have improved overall survival and progression-free survival compared to IDH wild-type tumours.^3–6^ IDH mutation status therefore appears to be closely linked to tumour behaviour and may be more important for prognostication than grade.^2^

To date, the literature suggests that IDH mutation is an early event in tumorigenesis and appears to be largely stable over time, though there have been reports of changes from wildtype to mutated IDH at tumour progression.^7–9^ Conversely, one study identified a subset of recurrent IDH mutant tumours with copy number alterations at the *IDH1* locus, leading to an IDH wild-type phenotype, a higher-grade tumour and a reprogrammed epigenome at tumour recurrence, resulting in a poor prognosis.^10^ Similarly, a case report on the multi-regional sampling of a patient with a grade 4 IDH mutant astrocytoma at primary and recurrent disease identified extensive mutational and copy number heterogeneity, including a loss of the clonal *IDH1* mutation in the recurrent tumour and poor outcome.^11^ Furthermore, IDH-mutated models are notoriously difficult to study both in vitro and in vivo, due to additional mutations or deletions that occur leading to alteration of the *IDH1* allele, and the low efficiency of xenograft formation, respectively.^12^ Therefore, a change in IDH status from mutant to wildtype may be secondary to altered tumour genetics, leading to increased aggressiveness and a poor prognosis.

The purpose of this study is to report on the institutional experience concerning the stability of IDH mutations in glioma treatment and tumour progression. To do this, we reviewed all glial tumours archived at our institution from 2009–2018, corresponding with our institution’s adaptation of IDH mutation testing after the initial discovery in 2008,^13^ and identified four patients with conflicting IDH mutation status longitudinally. These patients all had a loss of IDH mutation status as determined by our institution’s standardized practice of IHC testing of IDH mutations using formalin-fixed-paraffin-embedded (FFPE) tissue. In order to investigate these discrepancies in IDH mutation status, we performed methylation profiling including copy number variation, Sanger sequencing and ddPCR on longitudinal fresh frozen tissue samples and repeated IHC testing for IDH mutations using FFPE from all patients. All IDH mutation discrepancies were explained and resolved through multi-platform analysis. Our results support the concept that IDH mutation is a stable event throughout glioma evolution and highlight the role of accurate intraoperative tumour sampling and DNA methylome profiling as an adjunct diagnostic technique.

## Methods

### Patients and Clinical Samples

**Figure 1:**
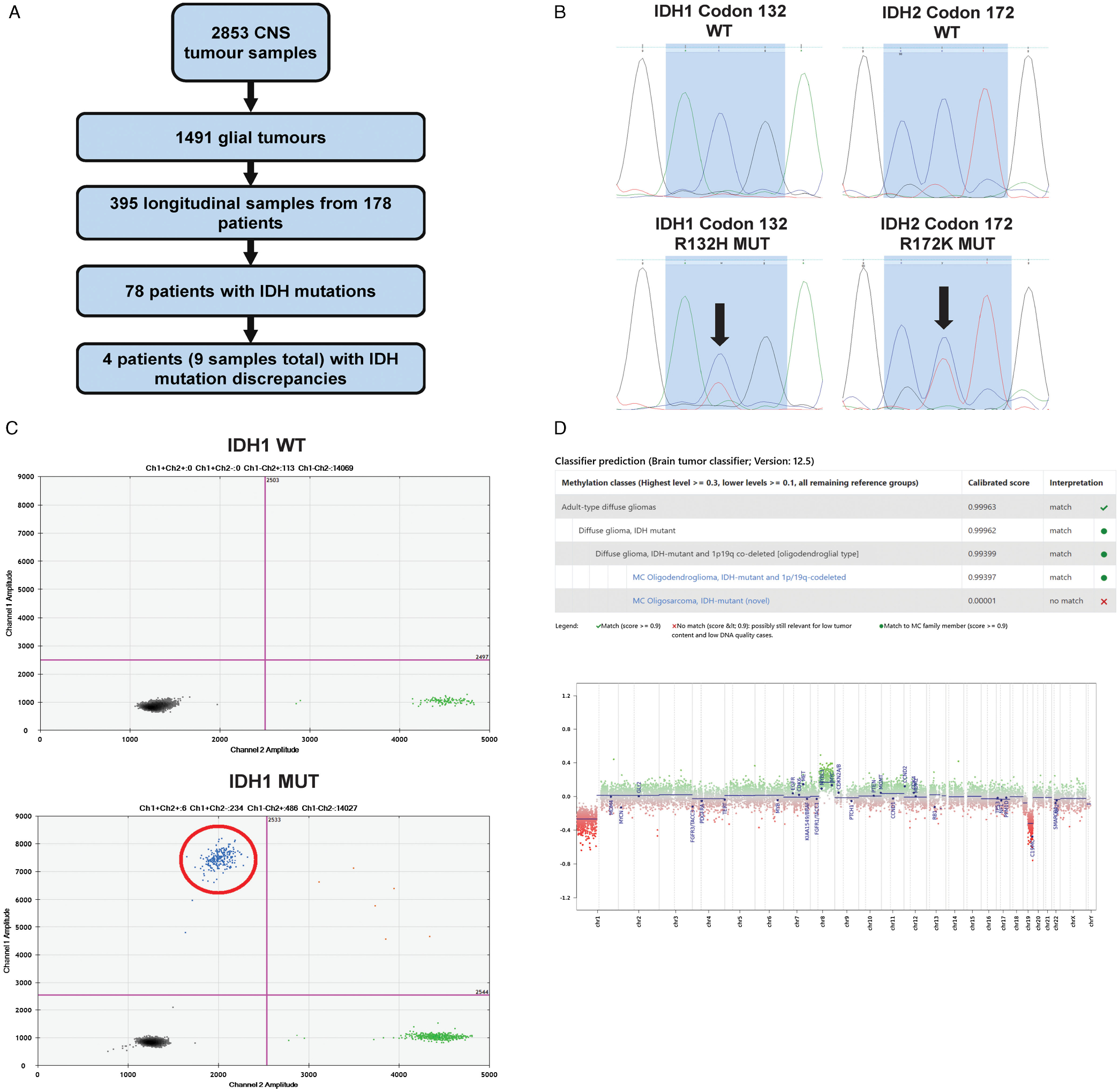
Investigating discrepancies in IDH mutation status in longitudinal glioma samples. Flowchart documenting the sample and patient review for the study. ***a***) Sanger sequencing of the *IDH1* and *IDH2* genes focusing on the regions directly surrounding codon 132 and 172, respectively. The presence of two peaks (arrows) demonstrates a heterozygous mutation in each of the mutated samples. ***b***) Droplet digital PCR identification of both an *IDH1* wt sample (above) and *IDH1* R132H mutation (below). Black dots (bottom left) represent double negative droplets, green dots (bottom right) represent IDH wt droplets, blue dots (top left) represent *IDH1* R132H mutant droplets, and orange dots (top right) represent double positive droplets. ***c***) DKFZ CNS methylation classifier example showing the calibrated scores with the final diagnosis and the copy number variation plot generated.

The study was approved by the University of Toronto research ethics board. Our brain tumour bank consisting of over 2500 CNS tumours catalogued from 2009 to 2018 was queried for glioma patients with matched longitudinal tumour samples (Fig. 1a). A total of 178 patients with longitudinal glioma tumour samples were identified and reviewed. This included 78 patients with IDH mutations. Four patients with IDH mutation discrepancies as documented from our institution’s routine *IDH1* R132H mutation testing using IHC were identified. This antibody test recognizes the *IDH1* R132H mutation which is present in approximately 90% of mutated cases but does not recognize other non-canonical *IDH1* or *IDH2* mutations.^14^ Fresh frozen tissue from each patient was collected at all available surgical timepoints (9 samples total, approximately 20 mg–25 mg tissue per sample). A neuropathologist reviewed all available tissue blocks for all 9 samples, and *IDH1* R132H IHC was repeated on IDH discrepant samples. Patient demographics, clinical information and pathology reports were collected and reviewed from electronic medical records. All diagnoses were based on the WHO 2021 CNS Guidelines.^1^

### DNA Preparation

All fresh frozen tumour samples underwent DNA extraction using the DNeasy Blood and Tissue Kit (Qiagen). The Qubit Assay Kit (Thermo Fisher Scientific) was used to quantify and measure the concentration of DNA. All kits were used according to the manufacturers’ instructions.

### IDH Mutation Testing

A total of 5 ng of tumour DNA were used from all 9 tumour samples for each method of IDH mutation testing. Gold standard Sanger sequencing was performed for both *IDH1* and *IDH2* genes containing the regions of interest involved in *IDH1* and *IDH2* mutations, respectively. Primers were designed in our laboratory to flank exon 4 of both *IDH1* and *IDH2* genes in order to identify the presence of important mutations.

*IDH1* forward primer: 5’ – TGG TGT ACT CAG AGC CTT CG – 3’

*IDH1* reverse primer: 5’ – AGT TGG AAA TTT CTG GGC CA – 3’

*IDH2* forward primer: 5’ – AAT TTT AGG ACC CCC GTC TG – 3’

*IDH2* reverse primer: 5’ – TGT GGC CTT GTA CTG CAG AG – 3’

*IDH1* R132H mutation testing was also performed using quantitative polymerase chain reaction (qPCR, Thermo Fisher) and droplet digital PCR (ddPCR, Bio-Rad) with a TaqMan SNP Genotyping Assay C_167891677_20 (Thermo Fisher).

#### Methylation Profiling and Copy Number Analysis

**Figure 2:**
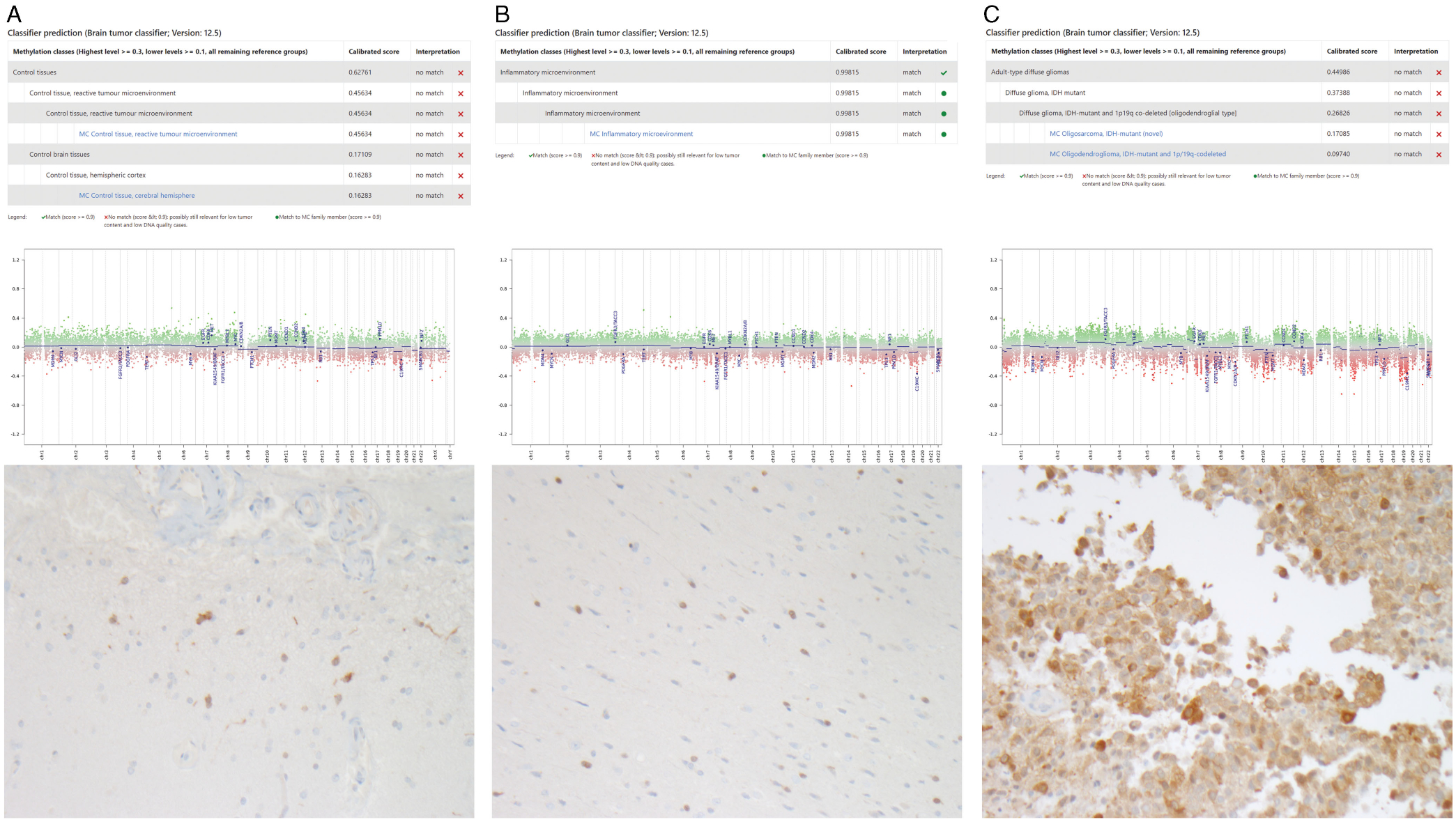
Integrated molecular and pathological diagnosis of IDH discordant samples from three patients. Each discordant sample has the DKFZ methylation classifier result (***a***), copy number variation plot (***b***) and repeated *IDH1* R132H antibody IHC testing of pathological tissue (***c***).

A total of 550 ng of tumour DNA from each sample was bisulphfite converted using the EZ DNA Methylation Kit (Zymo). The Infinium HumanMethylation450 BeadChip (Illumina) was then used to analyse the genome-wide methylation profiles of all 9 glioma samples. Once the raw (IDAT) files were obtained, these were uploaded to the DKFZ methylation profiling classifier at https://www.molecularneuropathology.org/mnp for methylation profiling, as shown in Figure 2.^15^ The DKFZ methylation classifier produces raw scores representing specific methylation classes. A calibration model then transforms the raw scores into calibrated scores, which represent an estimated probability measure of methylation class assignment, ranging from 0 to 1. An optimal calibrated score threshold for a single class is ≥ 0.9, and this value was used as the cut-off for all samples.^15^ Chromosomal copy number variation plots are created as part of the DKFZ methylation classifier result using the Bioconductor package conumee. Samples are compared to two sets of 50 control samples displaying a balanced copy number profile from both male and female donors.^15^ Standard copy number thresholds using the conumee package include gains or losses of ± 0.4, with less strict cut-offs of ± 0.2.^15^ Version 12.5 was used for all samples.

**Figure 3:**
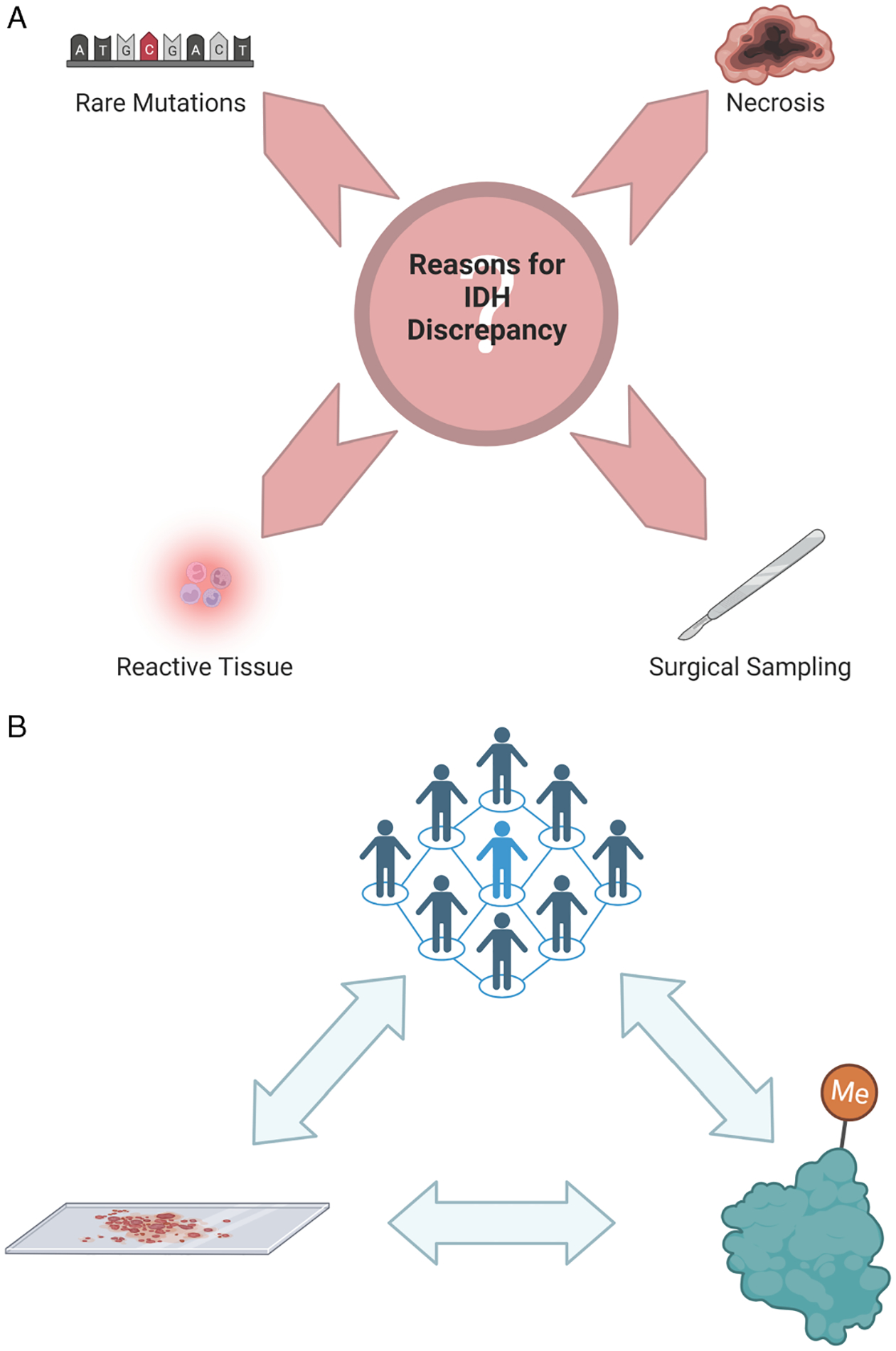
Reasons for IDH discordance and study conclusions. ***a***) Reasons for discrepancies in IDH mutation status over time. ***b***) Integrated approach between clinicians, histopathology and DNA methylation.

Figure 3 was created with BioRender.com.

## Results

A total of 178 glioma patients with longitudinal tumour samples were identified and reviewed. All patients had routine institutional testing of IDH mutation status by *IDH1* R132H antibody at time of tumour sampling. From these 178 patients, 78 patients had IDH mutations identified through IHC, and a total of four patients were found to have a mismatch in their IDH mutation status as documented from surgical pathology reports longitudinally (Fig. 1a). Table 1 summarizes the demographics and clinical course of all patients. Frozen tissue was collected from our institution’s tumour bank at all available surgical timepoints (minimum of 2 samples per patient, 9 samples total). All samples underwent methylation profiling including copy number variation and both Sanger sequencing and ddPCR testing of IDH mutation status in order to explore the discrepancies in the IHC-reported IDH status (Fig. 1b–d). All samples underwent additional pathological review, and three samples had repeat IHC performed on the most representative tissue available. Table 2 summarizes the initial histopathological and molecular findings of each tumour sample and the results of the additional analyses performed for this study.

Our results demonstrate that all four patients had IDH mutations present at recurrent surgical resection, and no patients had a change in IDH mutation status after treatment or during glioma evolution. The first patient was found to have a rare *IDH2* R172K mutation that was identified through our gene sequencing analyses (Fig. 1b) but would have been missed using only an *IDH1* R132H antibody on IHC. Our methylation profiling also identified the diagnosis of this patient as an IDH mutant 1p/19q codeleted oligodendroglioma. This patient, while identified during our review as having an IDH mutation discrepancy, also had a documented 1p/19q codeletion at time of initial surgery, which is mutually inclusive of having an IDH mutation.^1^ Therefore, this patient was originally treated as an IDH mutant oligodendroglioma despite the negative IHC testing and does not represent a case of IDH mutation discrepancy.

Of the remaining three patients, all had documented negative IHC testing for IDH mutation at tumour recurrence, despite their tumours initially demonstrating IDH mutations on IHC testing. DNA methylome profiling and methylation classification of these discordant samples identified a combination of perilesional tissue from surgical sampling that resulted in a mixture of low recurrent tumour content mixed with either normal brain tissue, reactive tumour microenvironment, or inflammatory microenvironment (Figure 2). Overall, this represented less than a 3% sampling error rate of all samples reviewed (9/395, Fig. 1a). All patients had copy number values for *IDH1* and *IDH2* genes < |0.1|, demonstrating that there were no copy number variations present in any sample. IHC for R132H *IDH1* mutations on these three discordant cases confirmed positive staining in all cases, with low tumour content in two and high tumour content in a subsequent tissue block that had previously not had IHC testing completed (Fig. 2).

Patient 2 (Fig. 2a) did not have a diagnostic match on the methylation classifier, with both control tissue and reactive tumour microenvironment the highest calibrated scores. This patient had a bland copy number plot suggesting non-neoplastic tissue, and IHC had positivity in only a small proportion of cells, demonstrating low tumour cell content. Patient 3 (Fig. 2b) had a methylation classifier match of 0.99 for inflammatory microenvironment and a bland copy number plot. Repeat IHC demonstrated positivity in a small proportion of cells, also demonstrating low tumour cell content. The final patient (Fig. 2c) did not have a methylation classifier match, although the highest calibrated score was in fact an adult-type diffuse glioma, with the second highest score being an IDH mutant 1p/19q codeleted diffuse glioma. The copy number plot for this sample was indicative of some tumour cells being present, with some losses observed at both 1p and 19q. IHC testing on a previously untested tissue block demonstrated positivity in a high proportion of cells.

These results support the hypothesis that IDH mutations are an early event in glioma tumorigenesis and remain unaltered throughout glioma evolution and treatment in the absence of major copy number alterations. Our research highlights the importance of accurate surgical sampling and the integrated role of DNA methylome profiling and copy number analysis as a confirmation of IDH status and its use in cases of diagnostic uncertainty.

## Discussion

In this study, we investigated patients with IDH discrepancies between multiple tumour samples collected longitudinally from routine clinical testing using IHC. This included three instances of an apparent loss of IDH mutation at recurrence and one case of a rare non-canonical *IDH2* mutation. In every case, we were able to identify a reason for the IDH mismatch and resolve the IDH mutation status, confirming the stability of IDH status longitudinally (Fig. 3a). All tumours exhibited an IDH mutation at recurrent surgical resection despite tumour treatment, evolution and recurrence, with no patients demonstrating copy number variations in IDH loci. Our data support the hypothesis that IDH mutation is an early event in glioma tumorigenesis and in the absence of genomic structural rearrangements, remains stable throughout the course of disease.

Previous studies have supported the concept that IDH mutation occurs early in gliomagenesis;^8,9^ however, multiple studies have found a subset of patients with changes in IDH mutation status at tumour recurrence. A study of 45 samples from paired low-grade astrocytomas and subsequent secondary high-grade gliomas found no changes in IDH mutation status.^8^ Another study of 51 lower-grade glioma patients that underwent multiple biopsies established a temporal timeline where IDH mutation precedes TP53 mutation or 1p/19q codeletion based on the proportion of patients that developed these mutations over time. A total of 41% of tumours that did not have TP53 or 1p/19q codeletions in initial samples went on to develop these in a subsequent biopsy, compared to only two patients (22%) that did not initially have IDH mutations that developed these over time.^9^ Another study examining 186 pairs of primary-recurrent GBMs from the EORTC 1542 study noted that three patients lost IDH mutation status at tumour recurrence.^16^ Efforts from the Glioma Longitudinal Analysis Consortium analysed longitudinal samples from 222 patients with gliomas and found that both IDH mutations and 1p/19q codeletions were not lost or gained in any patient during the surgical interval between samples.^17^

Few studies have explored the genetic underpinnings of apparent IDH mutation changes over time, but two studies independently found copy number alteration, potentially due to sub-clonal evolution, responsible for loss of IDH mutation at recurrence. One study reported copy number alterations in six recurrent gliomas at the *IDH1* gene, which led to a loss of IDH mutation at recurrence.^10^ This manuscript examined 50 patients with longitudinal tumour samples and found a 12% incidence of IDH mutation discrepancy over time.^10^ They reported that these tumours recurred as higher grade with reprogrammed epigenomes and decreased 2-hydroxyglutarate.^10^ A case report identified extensive mutational and copy number heterogeneity in a paired patient with a grade 4 IDH mutant astrocytoma, leading to the loss of IDH mutation at recurrence, hypothesized to be due to sub-clonal evolution and the evolution of a double-minute chromosome (extra-chromosomal circular DNA fragments frequently found in gliomas).^11^ Overall, we identified 4 patients out of 78 with longitudinally conflicting IDH mutation status, representing a 5% frequency of discrepancy, before further investigation.

It is important to note that any pathological or molecular analyses on brain tissue samples are only as good as the sample obtained. It is imperative that great care be taken as the operating surgeon to provide the most accurate biopsies and tumour samples for quick-section and for permanent pathology sections. This includes maximizing the use of image-guided sampling and providing permanent pathology and tumour bank samples in close proximity to the confirmed pathological quick-section sampling. In our study, three patients had previously documented negative IHC for IDH mutation at recurrence, suggesting a loss of IDH mutation over time. The Mib-1 (Ki-67) proliferation index and pathology report did note the presence of neoplastic cells in these specimens. DNA methylation profiling more accurately analysed these tissues and demonstrated suboptimal tissue samples including the presence of non-tumour cells identified as normal brain tissue, reactive tumour microenvironment and inflammatory tissue. Importantly, pathological review and repeat IHC testing did identify positive tumour cell staining for IDH mutation in all three samples.

Furthermore, we must also be aware of the limitations of the analyses performed on our tissue samples, including the sensitivity and specificity of the tests performed. A recent report on the R132H antibody for IHC testing compared to PCR testing identified a specificity of 100% and a sensitivity of 80%.^18^ The previously documented negative IHC testing despite the presence of neoplastic cells may indeed be due to false negatives, and three samples out of 78 patients with paired samples (3/156) represent a less than 2% false negative rate for our institution.

In conclusion, our study utilized multiple methods and approaches for determining IDH mutation status, most notably the addition of methylation profiling with copy number variation. Our results show that DNA methylome testing can reliably be used to determine the IDH mutation status of gliomas and provide diagnostic clarity. It can also be used to interrogate and explore IDH mutation discrepancies and determine if the tissue sample provided is less than ideal. As molecular approaches are continually advancing and becoming more commonplace, it is imperative that we integrate the pathology and molecular findings, just as we have integrated our neuro-oncology approach with a multidisciplinary team (Fig. 3b).

## Conclusions

All patients identified with discrepancies in their IDH mutation status were able to be resolved through methylation profiling. No patient experienced an IDH mutation alteration during the course of their treatment or at tumour recurrence. These findings support the hypothesis that IDH mutations occur early in gliomagenesis and in the absence of copy number variations at the IDH loci are stable throughout glioma evolution and treatment. This study highlights the importance of an interdisciplinary approach between the surgeon and the neuro-oncology team and an integrated approach of histopathology and novel diagnostic technologies including DNA methylome profiling for the accurate determination of IDH status and tissue diagnosis.

## Figures and Tables

**Table 1:** Patient demographics and clinical course

Patient	Age at Diagnosis	Sex	Presentation	Tumour Location	Number of Surgeries	Adjuvant Treatment	Overall Survival (years)
1	36	M	Seizure	Left frontal	4	Radiation, Temozolomide	5.3
2	42	M	Seizure	Right frontal	3	Radiation, Temozolomide, Lomustine	7.2
3	36	F	Incidental	Right frontal	3	Radiation, Temozolomide, Avastin	5.7
4	34	F	Seizure	Left frontal	3	Radiation, Temozolomide, Lomustine	5.2

**Table 2:** Tumour molecular testing

		Initial Pathological and Molecular Analysis				In-House Analysis				
Patient and Sample	Time in months	Histology	Grade	1p/19q status	IDH1 Testing by IHC	Sanger IDH1	Sanger IDH2	ddPCR IDH1	Methylation Testing	Reason for Discrepancy
1A*	0	Oligodendroglioma	3	–	WT	WT	R172K	WT	Oligodendroglioma, IDH mutation, 1p/19q codeletion	Non-canonical mutation (IDH2 R172K)
1B	160	Oligodendroglioma	3	codeletion	WT	WT	R172K	WT	Oligodendroglioma, IDH mutation, 1p/19q codeletion	
1C	186	Oligodendroglioma with treatment effect	3	–	WT	WT	R172K	WT	Oligodendroglioma, IDH mutation, 1p/19q codeletion	
1D	201	Oligodendroglioma with treatment effect and radiation necrosis	3	–	–	–	–	–	–	
2A*	0	Grade 4 astrocytoma	4	non-codeleted	MUT	–	–	–	–	Treatment effect / sampling error
2B	6	Recurrent grade 4 astrocytoma with treatment effect	4	–	MUT	R132H	WT	R132H	High-grade astrocytoma, IDH mutation	
2C	38	Recurrent grade 4 astrocytoma with treatment effect	4	–	WT	WT	WT	WT	No match – normal brain tissue with low tumour content / reactive tumour microenvironment	
3A*	0	Oligodendroglioma	3	codeletion	MUT	–	–	–	–	Inflammatory microenvironment / treatment effect
3B	111	Recurrent oligodendroglioma	3	–	MUT	R132H	WT	R132H	Oligodendroglioma, IDH mutation, 1p/19q codeletion	
3C	124	Gliotic brain with treatment effect, favoured reactive	4	–	WT	WT	WT	WT	Inflammatory microenvironment	
4A*	0	Oligodendroglioma	3	codeletion	MUT	–	–	–	–	Sampling error
4B	43	Recurrent oligodendroglioma	3	–	WT	WT	WT	WT	No match – adult-type diffuse glioma / IDH mutant 1p/19q codeletion	
4C	58	Oligodendroglioma with treatment effect	3	–	MUT	R132H	WT	R132H	Oligodendroglioma, IDH mutation, 1p/19q codeletion	

* Surgery performed at outside hospital.
